# 不正指令電磁的記録に関する罪における客体性要件について

**DOI:** 10.12688/f1000research.115029.1

**Published:** 2022-06-15

**Authors:** 髙良幸哉 髙良

**Affiliations:** 1 Faculty of Library, Information and Media Science, 1-2, Tsukuba-shi, Ibaraki-ken, 305-8550, Japan

**Keywords:** Coinhive, crimes related to electronic or magnetic records containing unauthorized commands, counterintentionality, unauthorized, cryptojacking

## Abstract

不正指令電磁的記録に関する罪（刑法168条の２および３）においては、客体性の要件として、当該コンピュータの利用者の意図に反すること（反意図性）および当該プログラム等が社会的に許容されないこと（不正性）が要求される。反意図性及び不正性の要件においてその意義あるいは考慮要素について、実務上は明確化されていなかった。本稿は、コインハイブ事件を素材に、文献調査と判例研究の方法で、不正指令電磁的記録に関する罪における反意図性と不正性の要件について検討する。加えてコインハイブ事件最高裁判決の意義と射程を明らかにするものである。

## Abstract (en)

The crime of electromagnetic records under unauthorized instruction (Article 168-2 and 3 of the Penal Code) requires that the program or the instruction code in question is against the intention of the computer user (counterintentionality) and socially unacceptable (unauthorized), as the requirements of objectivity. The significance or factors to be considered in the requirements of “counterintentionality” and “unauthorized” have not been clarified in practice. This paper examines the requirements of “counterintentionality” and “unauthorized” intent in crimes related to electromagnetic records with the Coinhive case as material, by means of a literature survey and case study. In addition, the significance and scope of the Supreme Court’s decision in the Coinhive case will be clarified.

## I. はじめに

2011 年 6 月 17 日に、第 177 回国会において「情報処理の高度化等に対処するための刑法等の一部を改正する法律」が成立してから 10 年余りが経過した。かかる法案は、「サイバー犯罪その他の情報処理の高度化に伴う犯罪及び強制執行を妨害する犯罪の実情に鑑み、情報処理の高度化に伴う犯罪に適切に対処するため、及びサイバー犯罪に関する条約の締結に伴い、処罰規定の整備や電磁的記録に係る記録媒体に関する証拠収集手続の規定の整備等を行い、並びに悪質な強制執行妨害事犯等に適切に対処するために処罰規定の整備等を行うべく刑法等を改正したもの」であり ^1^、本改正により、マルウェアなどを含むコンピュータ・ウイルスなどに関連する犯罪を規制する、不正指令電磁的記録に関する罪（刑法第 19 章の 2）として、不正指令電磁的記録作成等罪（同第 168 条の 2）、不正指令電磁的記録取得等罪（同第 168 条の 3）が新設されている。不正指令電磁的記録に関する罪においては、該当する電磁的記録が、人が電子計算機を使用するに際してその「意図に沿うべき動作をさせず、又はその意図に反する動作をさせるべき不正な指令を与える」もの（168 条の 2 第 1 項 1 号）であることが構成要件として要求されている。すなわち「反意図性」と「不正性」の要件である。

反意図性と不正性の認定基準については、従来必ずしも明確ではなく、裁判所の立場も不明確であった。ここで、反意図性と不正性をめぐっては近時注目すべき裁判例がある。いわゆるコインハイブ事件である。これはウェブサイト閲覧者の通信機器の中央処理装置 (CPU) の計算機能を一部用いることで暗号通貨のマイニングを行わせるプログラムを呼び出すプログラムコードを、ウェブサイトへ設置したことが不正指令電磁的記録の保管罪に該当するかが問題になった事案である。当該事案においては、原原審の横浜地裁、原審の東京地裁、および最高裁がそれぞれ異なる判断基準の下、判決を下しており、不正指令電磁的記録に関する罪の構成要件を明確化する上で参考になる。

本稿は、コインハイブ事件の各裁判例を分析・検討することを通して、不正指令電磁的記録に関する罪における反意図性と不正性の判断基準を明らかにすることを目的とするものである。

**方法**

本稿は、文献調査および判例研究の方法で行うものである。文献および判例は、 裁判所ウェブサイト、 Westlaw Japan、 Google Scholar、 CiNii Research、 LexisNexis、 Beck Online を使用し調査を行った。

## II. コインハイブ事件

### 1. 事案の概要 ^2^

本章においては、前述のコインハイブ事件における事案の概要及び、各裁判経過の内容について述べる。事案の概要は次の通りである。

ウェブサイト「Ｘ」を運営していた被告人は、Ｘ閲覧を通じて利益を得るため、平成 29 年 9 月 21 日、マイニングプログラムコードを提供しているサービスであるコインハイブ ^3^に登録し、提供されたプログラムコードに、被告人に割り当てられたサイトキーを記述したもの（本件プログラムコード）を、サーバコンピュータ上のＸ内に設置し、本件公訴事実の期間中、Ｘを構成するファイル内に蔵置して保管した。本件当時、一般の使用者に、ウェブサイトの収益方法として閲覧者の電子計算機にマイニングを行わせるという仕組みは認知されていなかったが、被告人は、Ｘに、閲覧中にマイニングが行われることについて同意を得る仕様を設けたり、マイニングに関する説明やマイニングが行われていることの表示をしたりすることなく、本件プログラムコードを保管していた。なお被告人は、本件プログラムコードにおいて、閲覧者の電子計算機の CPU 使用率を調整する値を 0.5 ^4^と設定していた。

### 2. 裁判経過

**・第一審（横浜地判平成 31 年 3 月 27 日判時 2446 号 78 頁）** ^5^ **: 無罪**

本件第一審は、反意図性を認定したものの、不正性を否定して無罪としている。

反意図性は、「個別具体的な使用者の実際の認識を基準とするのではなく，当該プログラムの機能の内容や機能に関する説明内容，想定される利用方法等を総合的に考慮して，当該プログラムの機能につき一般的に認識すべきと考えられるところ」を判断基準とする。

その上で、Ｘには本件マイニングプログラムについての一般的な認知や、マイニングについての同意を得る仕組みがないといった、上述の事実から、一般的に認知されず点から、「一般的なユーザーが認識すべきと考えられるものということはできない」として反意図性を認定する。

不正性については、「ウェブサイトを運営するような特定のユーザー及びウェブサイト閲覧者等の一般的なユーザーにとっての有益性や必要性の程度，当該プログラムのユーザーへの影響や弊害の度合い，事件当時における当該プログラムに対するユーザー等関係者の評価や動向等の事情を総合的に考慮し，当該プログラムの機能の内容が社会的に許容し得るものであるか否か」を判断基準とした上で、①「（運営者が得た利益が）ウェブサイトのサービスの質を維持・向上させるための資金源になり得るのであるから，現在のみならず将来的にも閲覧需要のある閲覧者にとっては利益とな」り、②「消費電力の増加，処理速度の低下等の影響が生じるが，その程度は広告表示プログラム等の場合と大きく変わることがな」く、「サイト閲覧中に限定され」ることを基礎に、本件プログラムコードが社会的に許容されていなかったとはいえないとして、不正性を否定した。

**・原審（東京高判令和 2 年 2 月 7 日判時 2446 号 78 頁）** ^6^ **: 破棄自判・有罪**

反意図性について、「当該プログラムの機能について一般的に認識すべきと考えられるところを基準とした上で，一般的なプログラム使用者の意思に反しないものと評価できるかという観点から規範的に判断されるべき」とし、第一審の検討については、規範的検討を行っていない点で判断手法には問題があるとする。その上で、本件プログラムコードについては、「プログラム使用者に利益をもたらさないものである上，プログラム使用者に無断で電子計算機の機能を提供させて利益を得ようとするものであり，このようなプログラムの使用を一般的なプログラム使用者として想定される者が許容しないことは明らか」として、反意図性を認定した結論においては第一審を支持している。

不正性については、「一般的なプログラム使用者の意に反するプログラムであっても，使用者として想定される者における当該プログラムを使用すること自体に関する利害得失や，プログラム使用者に生じ得る不利益に対する注意喚起の有無などを考慮した場合，プログラムに対する信頼保護という観点や，電子計算機による適正な情報処理という観点から見て，当該プログラムが社会的に許容されることがある」ことから、反意図性のある場合に処罰範囲を限定するための要件であるとする。

その上で、本件プログラムコードは、「その使用によって，プログラム使用者（閲覧者）に利益を生じさせない一方で，知らないうちに電子計算機の機能を提供させるものであって，一定の不利益を与える類型のプログラムといえる上，その生じる不利益に関する表示等もされていない」から、「プログラムに対する信頼保護という観点から」、社会的に許容できないする。

加えて、第一審が挙げた閲覧者の利益 (①) については、「意に反するプログラムの実行を，使用者が気づかないような方法で受忍させた上で，実現されるべきものでない」上、広告表示プログラムとの類似性(②) については、「広使用者のウェブサイトの閲覧に付随して実行され，また，実行結果も表示されるものが一般的であり，その点で，閲覧者の電子計算機の機能を閲覧者に知らせないで提供させる機能のある本件プログラムコードとは，大きな相違があ」るとして、不正性を否定する要素にはならないことを認めている。第一審は、コインハイブについての賛否があることを社会的許容性を肯定する評価に用いているが、この点についても否定をしている。

以上により、不正性を認定し、第一審を破棄し有罪とした。これを受けて弁護人が上告したのが本件である。

**・最（一）判令和 4 年 1 月 20 日（裁判所ウェブサイト掲載）** ^7^ **: 破棄自判・無罪**

不正指令電磁的記録に関する罪の保護法益および反意図性と不正性要件を設けた目的について「電子計算機において使用者の意図に反して実行される不正プログラムが社会に被害を与え深刻な問題」であり、「電子計算機において使用者の意図に反して実行される不正プログラムが社会に被害を与え深刻な問題となっていることを受け、電子計算機による情報処理のためのプログラムが、『意図に沿うべき動作をさせず、又はその意図に反する動作をさせるべき不正な指令』を与えるものではないという社会一般の信頼を保護し、ひいては電子計算機の社会的機能を保護するために、反意図性があり、社会的に許容し得ない不正性のある指令を与えるプログラムの作成、提供、保管等を、一定の要件の下に処罰するものである」ことであるとする。

反意図性については、「当該プログラムについて一般の使用者が認識すべき動作と実際の動作が異なる場合に肯定される」のであり、「一般の使用者が認識すべき」か否かは、「当該プログラムの動作の内容」、「プログラムに付された名称」、「動作に関する説明の内容」、「想定される当該プログラムの利用方法」などを判断の基底におくとする。

　不正性については、「電子計算機による情報処理に対する社会一般の信頼を保護し、電子計算機の社会的機能を保護するという観点から、社会的に許容し得ないプログラムについて肯定される」か否かを判断するのであり、「当該プログラムの動作の内容」、「その動作が電子計算機の機能や電子計算機による情報処理に与える影響の有無・程度」、「当該プログラムの利用方法」などが考慮要素となるとする。

以上の点から、「閲覧中にマイニングが行われることについて同意を得る仕様になっておらず、マイニングに関する説明やマイニングが行われていることの表示もなかった」のであり、「ウェブサイトの収益方法として閲覧者の電子計算機にマイニングを行わせるという仕組みは一般の使用者に認知されていなかった」といった事情から、本件プログラムコードを一般の閲覧者の「認識すべき」ものではなく、反意図性を肯定している。

一方で不正性については、「本件プログラムコードによるマイニングは、閲覧者の同意を得ることなくその電子計算機に一定の負荷を与え、これに関する報酬を閲覧者が取得することができないものであるのに、閲覧者にマイニングの実行を知る機会やこれを拒絶する機会が保障されていないなど、プログラムに対する信頼という観点から、より適切な利用方法等が採り得た」としつつも、「（上記の）保護法益に照らして重要な事情である電子計算機の機能や電子計算機による情報処理に与える影響は、Ｘ閲覧中に閲覧者の電子計算機の中央処理装置を一定程度使用することにとどまり、その使用の程度も、閲覧者の電子計算機の消費電力が若干増加したり中央処理装置の処理速度が遅くなったりするが、閲覧者がその変化に気付くほどのものではなかった」こと、「ウェブサイトの運営者が閲覧を通じて利益を得る仕組みは、ウェブサイトによる情報の流通にとって重要であるところ、被告人は、本件プログラムコードをそのような収益の仕組みとして利用したものである上、本件プログラムコードは、そのような仕組みとして社会的に受容されている広告表示プログラムと比較しても、閲覧者の電子計算機の機能や電子計算機による情報処理に与える影響において有意な差異は認められ」ないものとし、「事前の同意を得ることなく実行され、閲覧中に閲覧者の電子計算機を一定程度使用するという利用方法等も同様であって、これらの点は社会的に許容し得る範囲内といえる」として、本罪の保護法益に照らし、社会的許容性を認定する。またマイニング自体の社会的許容性も認めている。

以上の点から、「本件プログラムコードの動作の内容、その動作が電子計算機の機能や電子計算機による情報処理に与える影響、その利用方法」などを考慮しても、不正性は認定できないとして、無罪とした。

### 3. 判決の整理

コインハイブ事件をめぐる一連の判決において、論点となるのは反意図性および不正性についての判断基準である。不正指令電磁的記録に関する罪においては、その客体性要件である反意図性と不正性の認定基準が不明確であり、従前の裁判例においてもこの点を詳細に検討判断したものは見受けられない。上記3判決においては、次の点が注目できる。

・保護法益:規範的判断の基礎となる保護法益の理解

・反意図性:反意図性の判断における規範的評価

・不正性:考慮要素と判断の妥当性

以上、3 点において、コインハイブ最高裁判決を検討したい。その前提として、次章において、従来の議論を整理する。

## III. 不正指令電磁的記録に関する罪

### 1. 概要

不正指令電磁的記録に関する罪 ^8^は前述の通り、2011 年刑法改正により新設されたものであり、マルウェアやコンピュータ・ウイルスにかかる犯罪に対応するための犯罪類型である。本罪の新設以前においては、不正なプログラムである電磁的記録によってコンピュータが破壊あるいはそれにより業務が妨害された際に、器物損壊罪（刑法第 261 条）や公電磁的記録損壊罪（刑法第 258 条）、電子計算機損壊等業務妨害罪（刑法第 234 条の 2）が成立する場合を除けば、不正プログラムの作成、提供、供用、取得、保管の各段階は規制対象ではなかった。本罪成立より前では、例えば、ファイル共有ソフトを通じてウイルスファイルを送付し、他者のハードディスク内のファイルを使用できない状態にした事案につき、器物損壊罪で起訴したものがある ^9^。

本罪の保護法益は、「コンピュータ・プログラムがコンピュータに意図せざる不正な動作をさせるものではないことに対する信用・信頼」であり ^10^、社会的法益であると理解されてきた。法務省作成の解説においても、「「人が電子計算機を使用するに際してその意図に沿うべき動作をさせず、又はその意図に反する動作をさせるべき不正な指令」を与えるものではないという、電子計算機のプログラムに対する社会一般の者の信頼」 ^11^とされ、通説的には社会的法益として分類されるとともに、個人的法益に対する罪である電子計算機損壊等業務妨害罪あるいは公電磁的記録損壊罪の予備罪としての構成も否定されている ^12^。

個人的法益に対する罪の予備罪として構成することが否定された理由は、立案担当者の「個人情報を流出させるウイルスや電子メールを勝手に送信するウイルス、画像データなど権利・義務に関するものでない私電磁的記録を勝手に消去・改変するウイルスを作成、供用する行為が処罰対象に含まれないことになり、相当性を欠く」 ^13^との指摘にあるように、マルウェアやコンピュータ・ウイルスにかかる構成要件を不当に狭めることを避けるためであったと考えられる。マルウェアやコンピュータ・ウイルスは、「個々の電子計算機被害を与えるにとどまらず、それを超えて社会一般に重大な損害を与える」 ^14^のであって、例えば、これらプログラムの設置・送信が継続する間、国境を越えた被害の拡散の危険性も継続し、プログラムが発信元から削除されても、すでにかかるプログラムに感染した受信者から他のユーザーへの被害の拡散もあるなど、インターネットが普及した状況下における危険なプログラムの被害発生可能性等に鑑みれば、本罪の保護法益を社会的法益とする考え方は妥当である ^15^。また、かかる理解は本罪が 19 章の 2 として、文書等に対する「社会一般の信頼」を保護法益とする 16 章以下の偽造罪類型の後に位置している関係からも自然である。

なお、本罪は、「正当な理由がないのに」（正当な理由の不存在）、「人の電子計算機における実行の用に供する目的で」（目的犯）、客体となる電磁的記録を作成、提供 （168 条の 2 第 1 項）、供用（同第 2 項）、取得または保管 (168 条の 3) する行為が構成要件に該当する。本罪の客体は、「人が電子計算機を使用するに際してその意図に沿うべき動作をさせず、又はその意図に反する動作をさせるべき不正な指令を与える電磁的記録」（168 条の 2 第 1 項 1 号）および「（1 号の）不正な指令を記述した電磁的記録その他の記録」（同 2 号) ^16^ であり、反意図性および不正性は、当該電磁的記録が不正指令電磁的記録に関する罪における客体性の要件である。情報セキュリティの検査や情報漏洩防止のための技術が同時に犯罪にも利用可能なもの ^17^については、当該プログラムがコンピュータや電磁的記録を毀損しうるものであったとしても、不正指令電磁的記録に該当するかについては確定せず ^18^、主観的構成要件としての「不正な目的」とならび、適法に使用される当該プログラムを除外する要件となる。

### 2. 反意図性および不正性

　「その意図に沿うべき動作をさせず、又はその意図に反する動作をさせる」すなわち反意図性は、立法立案者解説 ^19^ や大コンメンタール ^20^ の記述によれば、社会一般の信頼を保護法益とする以上、その「意図」についても、そのような信頼を害するものであるか否かという点から規範的に判断されるべきであるとされる。この点、および法務省解説 ^21^ に「規範的」という文言は用いられておらず、「反意図」である場合の具体例が示されているのみである。

この点、大コンメンタールの記述と法務省解説の齟齬について、198 回国会において質問主意書 ^22^ が提出されている。本質問主意書への回答において、「意図」についても「そのような信頼を害するものであるか否かという観点から、すなわち、個別具体的な使用者の実際の認識を基準とするのではなく、当該プログラムの機能の内容や機能に関する説明内容、想定される利用方法等を総合的に考慮して、その機能につき一般に認識すべきと考えられるところを基準として、規範的に判断されるべきもの」 ^23^ であるとされ、「一般に認識すべき」ところを基準とした「規範的」判断が法務省の見解であることが示されている。かかる見解は、「一般に認識すべき」か否、すなわち一般人の認識可能性の有無を、単なる事実判断ではなく、法益侵害性を有するものか否かという価値判断を含ものである ^24^。

反意図性が認められない場合として、立法立案者解説においては、電子計算機の利用者が、「そのプログラムの指令によって電子計算機が行う基本的な動作については当然に認識している上、それ以外の詳細な機能についても、使用説明書に記載されるなどして、通常、使用者が認識しうる」市販のソフトウェアや、説明書が付与されていない場合であっても「当該ソフトウェアの機能は、その名称や公開されているサイト上での説明等により、通常、使用者が認識しうる」ようなフリーソフトの場合が挙げられ、ポップアップ広告についても「通常、インターネットの利用に随伴するものであることに鑑みると、そのようなものとして一般的に認識すべき」場合であるとする ^25^。いずれの場合も一般人の認識可能性が判断基準である。ただし、かかる文言だけでは、一般人が特に知ることができるか、広く周知されているかというある意味事実的判断のみで判断可能であるようにも読める。

情報リテラシーについては利用者において差があり、当該プログラムについて個々人としてみた場合、例えばエンジニアにおいて一般的に知りうる情報を、情報機器に疎い者はもちろん通常のユーザーですら知りえないことは当然にあることである。この点、日々開発されるプログラムについて通常人においては知りえず、常に「一般的に認識すべき」場合ではなく、反意図性が認められうるのであり ^26^、一般人の認識可能性のみを基礎とするのであれば、反意図性の範囲は規範的判断によって限定した意味を失してしまうであろう。仮に「一般人に認識すべき」プログラムであったとしても、当該プログラムが多大な負担を強いるのであれば、一般人がこれを望まないことは当然にありうる。かかる判断においては、実質的な負担の存否・軽重などに鑑み、どの程度の負担であれば一般に許容できるのか、つまりは、社会的許容性を観念せざるを得ないと思われる ^27^。

立法立案者の解説によれば、「「意図に反するか」否かの判断と「不正」か否かの判断は、別個の観点からなされるものであり、両者は必ずしも完全に重複するものではな」いとされ、前者が利用者にとって「一般に認識すべき」か、後者が「社会的に許容しうる」かが判断の際の観点となるとされるが ^28^、両者は重なりあうものであり、形式的な分類はともかくとして、実質的な判断においては区別困難となろう ^29^。

なお、コインハイブ事件については、全ての裁判経過において反意図性と不正性を認定する判断枠組みは維持されている。反意図性が認められる場合においては、多くの事例で当該プログラムが社会の信頼を害するといえるが、不正性の要件によって社会的に例外的に許容しうるものを除外することが、かかる要件の目的である ^30^ともいえる。

不正性判断において、社会的許容性を考慮するに際して、単に「有害性」のみを基準として判断することは立法段階で否定されており ^31^、「プログラムの客観的な性質だけで「不正」か否かを決することは難しく、理論的には、プログラムの客観的な性質に加えて、犯罪を行うために使用する意図の有無により処罰の対象となるか否かを判断する」 ^32^との言及に見られる通り、不正性判断においても「意図」あるいはそれを支える認識可能性の要素は排除されるものではない。「一般に認識すべき」動作や機能でないことは、本件最高裁の様に一般に認識できないほどの軽微な損害という観点からは、社会的許容性を認定する要素となる一方で、ユーザーの認識の外でマルウェア等が起動することは、ユーザーやネットワークへの潜在的なリスクという意味で不正性の認定要素 ^33^ともなりうる。

### 3. クリプトジャッキング

不正性判断において、国際的動向についても言及しておきたい。クリプトジャッキング (Cryptojacking) とは、他人のコンピュータを密かに利用して、暗号通貨をマイニングする行為である ^34^。本件最高裁判決に関する「WLJ 判例コラム第 254 号」においても、「その後、種々の態様のサイバー攻撃が登場し、「不正な指令」の範囲が拡張していく。膨大な数の他人のパソコンを「ボット」として使うことにより、サイバーアタックが実行されている。スパムメールやフィッシングメールが蔓延し、他人のパソコンを乗っ取りマイニングを行って利益を得る行為が世界中で問題視されている」との指摘がある ^35^。

ドイツ連邦刑事庁 (BKA) の報告書によれば、計算上、2017 年 10 月上旬時点で、TOP10 万サイトのうち約 220 サイトがコインハイブのスクリプトを搭載し、これらのサイトを合計すると、1 ヶ月に約 5 億人のユーザーの利用があるとされる ^36^。セキュリティウェア企業などの定義によればクリプトジャッキングに該当するとするものがみられる。なお、クリプトジャッキングについては、インストール型とウェブサイト閲覧型があり、コインハイブは後者である。前者はいわゆるマルウェアに分類されうる ^37^。コインハイブが 2019 年にサービスを停止した後、すでに多くのウェブサイトが、ウェブサイト閲覧型のクリプトジャッキングから撤退したもののなおも、複数のサイトにクリプトジャッキングのスクリプトを搭載しているとされる ^38^。

先のドイツの例でいえば、コインハイブのように javascript を用いて、データ自体の変更を伴わないようなプログラムの場合には、データの変更に関する規定（ドイツ刑法 303a 条）の適用はない。また、他者のコンピュータへの介入について、DDoS はコンピュータの妨害（ドイツ刑法 303b 条）の対象となるが、ウェブサイト閲覧型のクリプトジャッキングは処理の中断を伴わないため対象とはならない ^39^。

なお、諸外国の動向を見るに、現在のところ、ウェブサイト閲覧型クリプトジャッキングについてのコインハイブ事件と同様の事案は見受けられず ^40^、マルウェアを用いたクリプトジャッキングについては複数の検挙事案がみられるところである ^41^。ウェブサイト閲覧型のクリプトジャッキングサービスのプライバシーリスクあるいは、暗号通貨自体が犯罪利用リスクの存在については問題となりうるが、かかるサービスを契約し利用する者に可罰的な違法性まで存するのかについては、議論が成熟していないのが現状であろう。

とはいえ、クリプトジャッキングが許容しえない損害を利用者あるいは社会全体に与えうるのだとすれば、これを国家として容認するには当然に問題がある。当該国家の住民対象のウェブサイトであれば、合法的に他者の CPU を利用することができるとの誤ったメッセージを与え、サイバー攻撃の誘引につながる危険性もあり得る。コインハイブにかかるプログラムコードが不正指令電磁的記録に該当するかどうかとは別論、クリプトジャッキング自体を明確に「適法」であるということは困難である。

## IV. コインハイブ事件最高裁判決の検討

本件最高裁判所は、反意図性を肯定しつつ不正性を否定して、有罪判決としていた原審東京高裁判決を破棄し、被告人を無罪とした。本章においては、最高裁判所における、不正指令電磁的記録に関する罪の保護法益、反意図性および不正性の判断についての見解について検討を行う。

**・保護法益について**

まず、反意図性および不正性の判断を保護法益を侵害するか否かの規範的判断によって行うのであれば、まず最高裁が本罪の保護法益をいかに理解しているかに言及する必要がある。ここでは、電子計算機で用いられるプログラムが、「意図に沿うべき動作をさせず、又はその意図に反する動作をさせるべき不正な指令」を与えるものではないという「社会一般の信頼」とするように、立法時からの理解が維持されている。この点、「ひいては電子計算機の社会的機能を保護」に言及する点、かかる社会的機能を重視した判断であり、「抽象的な信頼にとどまらないこのような機能自体、即ち電子計算機による適正な情報処理という具体的状態自体への着目は不正指令電磁的記録不正指令電磁的記録概念の要件解釈の重要な指標」 ^42^ となりうるとの指摘がある。立法担当者においても、当初より電子計算機の社会的機能の重要性については言及されていたところである ^43^。もちろん最高裁が「社会一般の信頼」保護の結果ないし反射的効果として保護されるものとして、「電子計算機の社会的機能」を明示した点には意義がある。ただし、本件最高裁は、「閲覧者の電子計算機の機能や電子計算機による情報処理」への影響の社会的許容性を問題としているのであり、電子計算機自体の社会的機能ではなく、生じうる損害への社会的評価に重点を置いている様に思われる。「電子計算機の社会的機能」への影響を重視するのであれば、不正性を認定する方向に傾くとも考えられよう。

**・反意図性について**

反意図性については、「一般の使用者が認識すべき動作と実際の動作が異なる場合に肯定される」とし、一般の認識可能性を判断基準としつつ、文言上は一般人の認識と現実の齟齬を判断している事実的判断であるようにも見える ^44^。一般の使用者が「認識すべき」か否かを判断基準とする点においては、一審からの一貫した判断であるが、「規範的」との文言が反意図性判断においては第一審と最高裁判決においては用いられていない。高裁判決は「規範的判断」に言及し、立法立案者の見解に立つことが明確であることと相違する。

この点、先の保護法益についての文言である「意図に沿うべき…保護するために、」という文言は、文脈上、反意図性と不正性双方にかかるものであり、反意図性判断においても、単なる認識と現実の齟齬といった事実的判断だけではなく、法益侵害性についての判断を行うことが前提であろう。本件が、ある種の事実的判断のみで反意図性が認定可能であるのは、本件が法益侵害性についての価値判断を行うまでもなく、反意図性が認定できる事案であったと思われる。本件において、「不正性」判断において規範的評価の中心がおかれているとはいえ、本件最高裁が反意図性の判断における規範的判断を放棄したとまではいえないであろう。

**・不正性について**

第一審及び原審において反意図性判断の要素であった「閲覧者の同意」「閲覧者にマイニングの実行を知る機会やこれを拒絶する機会」の保障といったものが、不正性判断の段階で検討されており、この点、反意図性と不正性は不可分であるとの私見によれば妥当な判断であると思われる ^45^。不正性については、原審において「閲覧者に利益を生じさせない一方で一定の不利益を与えるものである上、不利益に関する表示等もされない」点で、「閲覧者の電子計算機を、閲覧者以外の利益のために無断で使用する点を基礎に 不正性、本罪の保護法益である、「社会一般の信頼」保護という原則により依拠したものであるといえる。

不正性判断において、「社会一般の信頼」を侵害する危険性があるか否かを判断基準にすることについては、異論はない。しかし、同意を得ずに他者のコンピュータに介入操作するクリプトジャッキングの潜在的な危険性に鑑みれば、「社会一般の信頼」を害する危険性は少なくない。「本件プログラムコードの動作の内容であるマイニング自体は、仮想通貨の信頼性を確保するための仕組みであり、社会的に許容し得ないものとはいい難い」ことは、暗号通貨のマイニングによって報酬をえることそのものが適法であるのは当然のことであるが、かかる閲覧者の同意を得ない手法が「社会一般の信頼」を害しないといえるかには疑問が残る。本件最高裁の結論自体には異をとなえるものではないが、理由付けとしては、損害が軽微ゆえに、本件プログラムコードの掲載自体の社会的許容性あるとする方が妥当だったのではなかろうか。

また、社会的許容性を判断するにあたり、そもそも広告表示プログラムはその動作が閲覧者に秘匿されるものではなく、そのウェブ利用時の付随性も一般的に知られ、当該プログラムによる動作もかかる広告を画面表示させるにとどまる ^46^。一方で、マイニングプログラムは閲覧者の認識なくCPU を利用した計算処理を行った上、継続的かつ一方的に処理能力を奪い、電力を消費する。その上、計算結果の抜き取りについても本人の同意なく行われる。当該プログラムが与えうる侵害の危険性と、個人領域への干渉は小さくない。本件行為対応が社会的に許容される範囲に収まるとしても、マイニングプログラムにおいては潜在的なリスクが存する。その点を過小評価しているのではないかとの評価はなしうるところである。

## V. おわりに

以上、本稿においては不正指令電磁的記録に関する罪における反意図性と不正性を中心に若干の検討を行った。なお、コインハイブ事件に関して、本稿においては刑法構成要件の観点で検討を行ったが、コンピュータ・プログラムへの規制は研究開発への過度の制約となりえ、また、これにより表現の自由の侵害の危険性もあるものである。この点、本稿で挙げた評釈においても憲法的論点から論じる者が少なくない ^47^。この点、今後さらなる検討を行いたい。

結びに変えて、本稿で扱ったコインハイブ事件の射程と意義を述べたい。本稿は従来明らかでなかった反意図性と不正性について最高裁判所がはじめて判断した事案であり、今後の本罪にかかる事案について参考になるものである。ただし、反意図性は事実的判断、不正性については規範的判断であると、その判断方法について明確に区別したものとまでは言えないと考える。反意図性が認められる場合の処罰範囲を限定するものとして不正性の要件があり、反意図性判断が不正性判断の前段階におかれることが明示された点は評価できる。私論であるが、裁判所が反意図性判断における規範的判断を放棄していないと考えるのであれば、(1) 反意図性にかかる事実判断、(2) 反意図性にかかる規範的判断、(3) 不正性判断との流れで検討がなされ、処罰範囲を限定していくとも考えられる。ただし、判例における判断基準については、今後事案の蓄積を要し、本稿において断言することは難しい。

なお、付言すれば、プログラムを実装するに際し、情報流通の国境がなくなり、データプライバシーが重視される昨今においては、プライバシー・バイ・デザインを意識して技術の企画から実装までの各段階においてプライバシーへの尊重が求められるのであり、利用者や閲覧者の同意を得る仕組みを組み込んだ上で技術実装がなされることが望ましいことはいうまでもない。

## データ可用性

本論文は法学の論文であり、「生データ (raw data)」は存在しない。書籍や論文として公開されている学説、判例等に基づいている。

## Footnotes

^1^
第 177 回国会法務大臣による趣旨説明。第177回国会法務委員会第 13 号（平成 23 年 5 月 25 日） （江田五月）。

^2^
事案の概要および、最高裁判旨については、 裁判所ウェブサイトを参照。

^3^
最高裁の認定事実（前掲注 2）参照）によれば、「その内容は、登録したウェブサイトの運営者（以下「登録者」という。）に対し、ウェブサイト閲覧者が閲覧中に使用する電子計算機の中央処理装置に同閲覧者の同意を得ることなく仮想通貨 Monero （モネロ）の取引台帳へ取引履歴を追記する承認作業等の演算を行わせ、その演算が成功すると、報酬として仮想通貨の取得が可能になるというマイニングを実行するプログラムコードを取得するためのプログラムコードを提供し、報酬の 7 割を登録者に分配し、 3 割をコインハイブチーム側が取得するというもの」である。コインハイブ自体は 2019 年にサービスを終了している。

^4^
裁判所認定事実（前掲注 3）参照）によれば、「マイニングを実行すると、閲覧者の電子計算機の消費電力が若干増加したり中央処理装置の処理速度が遅くなったりするが、極端に遅くはならず、これらの影響の程度は、閲覧者が気付くほどではなく、また、一般的なウェブサイトで広く実行されている広告を表示するプログラム（以下「広告表示プログラム」という。）と有意な差異はなかった」とされる。そのため、個々のサイト閲覧者が被る実害自体は軽微であった。

^5^
第一審の解説及び評釈また先行研究としては、以下のようなものがある。板倉陽一郎「解題 コインハイブ事件[横浜地裁平31.3.27 判決]」 Law & Technology 85 号 (2019) 15-19 頁、高木浩光「コインハイブ事件で否定された不正指令電磁的記録該当性とその論点[横浜地裁平成 31.3.27 判決]」Law & Technology 85 号 (2019) 20-30 頁、永井善之「判批」法セ増（新判例解説Watch) 26 号 (2020) 187-190 頁。

^6^
原審の解説及び評釈としては、以下のようなものがある。品田智史「判批」法セ 787 号 (2020) 134 頁、永井善之「不正指令電磁的記録概念について」金沢法学 63 巻 1 号 (2020) 79-146 頁、三重野雄太郎「不正指令電磁的記録の解釈と該当性判断枠組:コインハイブ事件を素材に」社会学部論集 71 号 (2020) 127-148 頁、白鳥智彦「判批」警察学論集 73 巻 9 号 (2020) 206 頁、木下昌彦「コンピュータ・プログラム規制と漠然性故に無効の法理（上）コインハイブ事件を契機とした不正指令電磁的記録に関する罪の憲法的考察」NBL1181 号 (2020) 4-12 頁、木下昌彦「コンピュータ・プログラム規制と漠然性故に無効の法理（下）コインハイブ事件を契機とした不正指令電磁的記録に関する罪の憲法的考察」NBL1182 号 (2020) 39-50 頁、西貝吉晃「技術と法の共進化を企図した法解釈の実践: コインハイブ高裁判決を素材に」法セ 792 号 (2021) 40-46 頁、板倉陽一郎「解題 コインハイブ事件控訴審」Law & Technology 91 号 (2021) 40-45 頁、高木浩光「コインハイブ不正指令事件の控訴審逆転判決で残された論点」Law & Technology 91 号 (2021) 46-57 頁、永井善之「判批」法セ増（新判例解説 Watch) 27号 (2021) 153 頁、岡田好史「判批」刑事法ジャーナル 68 号 (2021) 159-164 頁、小田啓太=西貝吉晃「アプリ開発の実務を踏まえた不正指令電磁的記録に関する罪の一考察: コインハイブ事件を契機として」千葉大学法学論集 36 巻 (2021) 1 号 105-136 頁。

^7^
本稿執筆時点において、解説として、永井善之「判批」 新・判例解説 Watch 刑法 No.176 https://www.lawlibrary.jp/pdf/z18817009-00-071762137_tkc.pdf (2022-4-4参照）、コインハイブ事件にコメントするものとして、前田雅英「判批」WLJ判例コラム 254 号 (2022) (2022WLJCC006)、河津博史「判批」銀行法務 21 881 号 (2022) 70 頁がある。なお、本稿の執筆後、今井猛嘉 「判批」法教 500 号 (2022) 33-39 頁、西貝吉晃「判批」法セ 808 号 (2022) 46-55 頁に接した。

^8^
なお、不正指令電磁的記録に関する罪の立法経緯や解説については、立案担当者の解説である杉山徳明=吉田雅之「 『情報処理の高度化等に対処するための刑法等の一部を改正する法律』について（上） 」法曹時報 64 巻 4 号 (2012) 64 頁以下を参照。

^9^
いわゆる「イカタコウイルス事件」東京地判平成 23 年 7 月 20 日判タ 1393 号 366 頁。

^10^
山口厚「サイバー犯罪に対する実体法的対応」ジュリスト 1257 号 (2013) 18 頁、嶋矢貴之「第 19 章の 2 　不正指令電磁的記録に関する罪」西田典之他編『注釈刑法 第 2 巻』（有斐閣、2016) 545 頁ほか。

^11^
杉山=吉田・前掲注 8) 65頁。

^12^
法務省「 いわゆるコンピュータ・ウイルスに関する罪について 」(2012) 1 頁 http://www.moj.go.jp/content/001267498.pdf(2022-4-4参照）。

^13^
杉山=吉田・前掲注 8) 66 頁。

^14^
杉山=吉田・前掲注 8) 66 頁。

^15^
この点、法益の抽象化によって事実上の処罰の早期化につながるとの批判がある。渡邊卓也『ネットワーク犯罪と刑法理論』（成文堂、2018） 266 頁以下。

^16^
2 号類型については、「人が電子計算機を使用するに際してその意図に沿うべき動作をさせず、又はその意図に反する動作をさせるべき不正な指令を与える」ものとして実質的には完成しているものの、そのままでは電子計算機において動作をさせる状態にないものをいうとされ、不正な指令を与えるプログラムコードを記述した電磁的記録や紙媒体がこれに該当しうる。法務省文書・前掲注12) 5頁。なお、 2 号の客体は「プログラムの機能ないし構造上、「不正指令」を与えるものとして設計されているもの」に限定すべきであるとの主張もある。石井徹哉「いわゆる「デュアル・ユース・ツール」の刑事的規制について（中） 」千葉大学法学論集 26 巻 4 号 (2012) 17 頁以下。

^17^
いわゆるデュアルユース。石井徹哉「いわゆる「デュアル・ユース・ツール」の刑事的規制について（上） 」千葉大学法学論集 26 巻 1-2 号 (2012) 66 頁。

^18^
例えば、ハードディスク内のファイルを全て消去するプログラムが、その機能を適切に説明した上で公開されるなどしており、ハードディスク内のファイルを全て消去するという動作が使用者の「意図に反する」ものでない場合は、処罰対象とはならない。法務省文書・前掲注 12) 4 頁。

^19^
杉山=吉田・前掲注 8) 。

^20^
立法立案者の一人の執筆による。吉田雅之「第 19 章の2　不正指令電磁的記録に関する罪」大塚仁他編『大コンメンタール刑法第 8 巻（第三版） 』（青林書院、2014） 345 頁。

^21^
法務省文書・前掲注 12)。

^22^
松平浩一「不正指令電磁的記録に関する罪の解釈に関する質問主意書」（令和元年 6 月 21 日提出 質問第 294 号）。

^23^

「衆議院議員松平浩一君提出不正指令電磁的記録に関する罪の解釈に関する質問に対する答弁書」（令和元年 7 月 5 日受領　答弁第二九四号）。吉田・前掲注 20) 345 頁に同旨。

^24^
この点、「現実にその機能を認識したらその実行を許していたか?という判断も「べき」か否かの規範的判断に混入しうる点で処罰範囲が恣意的に決定されうる」 との危惧も指摘される。西貝吉晃「不正指令電磁的記録に関する罪の解釈論」罪と罰 58 巻 3 号 (2021) 21 頁。

^25^
杉山=吉田・前掲注 8) 71 頁参照。この点「なぜ一般に認識「すべき」なのかは判然としない。同広告を鬱陶しく感じるものが相当数いることが容易に想定できる以上、反意図性を定型的に否定するのは難しい」との批判もある。西貝・前掲注 24) 22 頁。

^26^
永井・前掲注 5) 189 頁に同旨。

^27^
永井（金沢法学） ・前掲注 7) 103 頁は「結局これは、反意図性についても不正性についても、その肯否の判断に際しては使用者一般により、即ち社会的に許容されるものであるかが基準とされざるをえないためであるように思われる」とする。

^28^
杉山=吉田・前掲注 8) 83 頁参照。

^29^
「意図に反する」か否かの判断と「不正」か否かの判断は連動すると解するのが自然であるとの見解として、渡邊・前掲注 15) 269 頁。

^30^
吉田・前掲注 20) 346 頁参照。この点、左記吉田の大コンメンタールにおける記述は、「「意図に反する動作をさせる」の解釈を保護法益に照らして信頼を害するものに限定」しており、「「意図に反する動作をさせるものなら」その時点で「信頼を害するものとして……当罰性がある」という当然のことを述べたに過ぎない」とする見方もある。高木・前掲注 5) 26 頁。

^31^
「 法制審議会刑事法（ハイテク犯罪関係）部会第 3 回会議議事録 」（平成 15 年 5 月 15 日開催） https://www.moj.go.jp/shingi1/shingi_030515-1.html(2022-4-4 閲覧）。

^32^
経済産業省「サイバー刑事法研究会報告書「欧州評議会サイバー犯罪条約と我が国の対応について」」21 頁。

^33^
この点、西貝・前掲注 24) 22 頁以下は、サイバー犯罪条約準拠の情報セキュリティ（CIA=機密性、完全性及び可溶性）観点から、CIAを侵害するか否かの観点から不正性要件、保護法益の理解の修正を検討している。

^34^
これをサイバー犯罪の一種であるとする記述もみられる。Interpol. “ Cryptojacking”. https://www.interpol.int/Crimes/Cybercrime/Cryptojacking （2022-04-04閲覧）。ただ、当該記述においてはインストール型を想定してる。

^35^
前田・前掲注 7) 5 頁。

^36^
Rainer Franosch. “Sicherheit in einer offenen und digitalen Gesellschaft”. BKA Herbsttagung 21, 22. November 2018, 2018. p.5.

^37^
例えば、Norton「 クリプトジャッキングとは?その基本から撃退方法まで 」ノートンウェブサイト、https://jp.norton.com/internetsecurity-general-security-cryptojacking.html（2022-4-4閲覧）参照。

^38^
この点、Said Varlioglu et al. “Is Cryptojacking Dead After Coinhive Shutdown?”. 2020 3rd International Conference on Information and Computer Technologies (ICICT). 2020, pp. 385 - 389 参照。

^39^
Franosch. op.cit. p. 5.

^40^
Google Scholar および LexisNexis 、Beck-Online の各文献検索サービスを用いて調査を行った。

^41^
例えば、ルーマニアの犯罪組織に属する被告人らが、共謀の上、電信詐欺、加重 ID 窃盗、マネーロンダリングへの関与、マルウェアを用いた暗号通貨のマイニングを行った事案につき合衆国において有罪となった事案がある。United States v. Nicolescu, 17 F.4th 706.

^42^
永井・前掲注 7) 3 頁。

^43^
杉山=吉田・前掲注 8) 65 頁。

^44^
永井・前掲注 7) 3 頁。この点、左記では、第一審についてもプログラムの機能との齟齬という事実的判断に立つとする。

^45^
原判決の様に、「反意図性につき形式的、事実的な判断ではなく、より実質的な規範的判断をなすとすると、それにより不正性要件の判断の先取りないし、それとの一体化となる」とし、本件最高裁の判断はこれを回避しうるとの見解もある。永井・前掲注7) 3頁以下。

^46^
仮に他の機能が当該広告表示プログラムに付随している場合においては個別の機能や動作において社会的に許容されるか否かを判断する必要があろう。

^47^
例えば、木下・前掲注 6)。

