# Supplementary material for: The objectivity requirement in crimes related to electronic or magnetic records containing unauthorized commands
Source: F1000Res. 2022 Jun 15;11:657. [Article in Japanese] [Version 1] doi: 10.12688/f1000research.115029.1 (PMC9253657; doi:10.12688/f1000research.115029.1)
Supplement: Peer review history

# Peer review: Reviewer response for version 1

**DOI:** 10.5256/f1000research.126781.r140927

**Published:** 2022-07-07

**Authors:** Ko Shikata

**Affiliations:** 1 Faculty of Law, Chuo University, Tokyo, Japan

本稿は、刑法の不正指令電磁的記録に関する罪の客体要件について、本罪の著名事件であるコインハイブ事件に対する最近の最高裁判決の評価を中心に検討したものである。本稿のような判例の評価という規範的な判断に関する客観性は、自然科学的な意味での客観性ではなく、多方面の視点や価値観を参照して得られるものと言える。その意味で本稿の特徴は、本判決の判例評釈の多くが指摘している技術開発の自由の視点を紹介するのはもちろん、他の評釈ではあまり顧みられてこなかった一般ユーザーの視点や、コインハイブというサービスやその技術的基礎となっているクリプトジャッキングに対する海外の視点をも紹介し、客観的でバランスのとれた評釈がなされている点にあるといえる。

 本罪の客体である不正指令電磁的記録の要件に関する論点としては、本稿も示しているように、一般に、本罪の保護法益、いわゆる反意図性要件、不正性要件が挙げられるが、前2者については論者による評価の差は比較的小さい。論者により評価が分かれ、本事件でも第一審・最高裁と高裁とで見解が分かれたのは不正性要件であった。

 反意図性要件は広く理解され本罪の処罰範囲を広げ得るものだけに、本罪が過剰な規制とならないためには不正性要件が適切な限定をかける機能を果たす必要があるのは、他の多くの評釈が指摘しているとおりである。他方で、本件をはじめとする一連のコインハイブ関連事件には、自己の端末の空き容量を知らない間に利用された多数の一般ユーザーが存在する。一人一人のユーザーにしてみると、その受けた影響は確かに被害と認識できない程度に軽微なものである。しかし、現在のインターネット空間においては極めて多数の者を相手に瞬時に行動を起こすことができ、個々人には微小な影響しか与えないプログラムであっても、ネットワークを通じれば多数の者の端末をその知らない間に利用して、全体としては大規模な利益をあげることができる可能性がある。個別の端末に対する影響を中心的な考慮要素とした本判決の枠組みでは、例えばボットネットを用いて個々のユーザーに対しては本件と同様に至極軽微な影響しか与えないが、数千、数万の一般ユーザーの端末を勝手に利用することによって莫大な利益をあげるような活動が許されることになりかねないのではないかという疑念がぬぐい切れない。

 このように考えると、我が国法体系が保護すべき情報セキュリティには、サイバーセキュリティ基本法第2条が示しているように、個々の端末のセキュリティだけでなく「情報通信ネットワークの安全性及び信頼性」が含まれるべきであり、本罪の保護法益である「電子計算機のプログラムに対する社会一般の者の信頼」にも情報通信ネットワークが公正に利用される信頼性の観点が含まれるべきではなかろうか。したがって不正性要件の判断においても、個々の端末への影響だけでなく情報通信ネットワークの信頼性・公正性を考慮して判断されるべきものであろう。

 査読者と同様の問題意識からであるかは分からないが、本稿は、「ユーザーの認識の外でマルウェア等が起動することは、ユーザーやネットワークへの潜在的なリスクという意味で不正性の認定要素ともなりうる」ことを指摘し、一般ユーザーや情報通信ネットワーク全体の信頼性に通ずる視点を示している。さらに、コインハイブに対する国際的評価やウェブサイト閲覧型のクリプトジャッキングの技術的危険性についても指摘している。

 その上で本稿は、本判決について、本件に使われた「当該プログラムが与えうる侵害の危険性と、個人領域への干渉は小さくない。本件行為対応が社会的に許容される範囲に収まるとしても、マイニングプログラムにおいては潜在的なリスクが存する。その点を過小評価しているのではないかとの評価はなしうるところである」と結論付けている。

 このように、本稿は、他の多くの評釈が指摘する技術開発の自由に十分配意しつつも、一般ユーザーや情報通信ネットワークの信頼、国際的社会の視点といった幅広い視野に立って、客観的で妥当な結論に達しているものと評価することができる。

（該当する場合は要回答）統計分析および解釈は適切ですか。

対象外（統計を使っていない）

本研究は明確かつ正確に提示されたものであり、最新の文献を引用していますか。

はい

方法と分析について第三者による再現が可能となるよう十分な詳細が提示されていますか。

はい

結果の基礎となるソースデータはすべて入手可能で再現性を十全に保証していますか。

はい

結論は結果により妥当な裏付けを得ていますか。

はい

研究設計は適切で学術的価値がありますか。

はい

Reviewer Expertise:

刑事法学、社会安全政策論

I confirm that I have read this submission and believe that I have an appropriate level of expertise to confirm that it is of an acceptable scientific standard.

# Response

**Published:** 2022-08-27

**Authors:** Kouya Takara

中央大学　四方光先生

 この度はお忙しい中、査読をお引き受けいただきありがとうございます。

 近日中に、他の査読者の先生方からのご指摘も踏まえ、ver. 2を投稿予定です。

 よろしくお願い申し上げます。

 髙良幸哉

# Peer review: Reviewer response for version 1

**DOI:** 10.5256/f1000research.126781.r140924

**Published:** 2022-07-05

**Authors:** Yoshifumi Okada

**Affiliations:** 1 School of Law, Senshu University, Tokyo, Japan

本論文では、暗号資産をマイニングするスクリプトの利用を巡り問題となった不正指令電磁的記録に関する罪の成立要件、とりわけ反意図性と不正性の関係について、最高裁として初めて判断を示した判例をもとに明らかにし、その判断基準について検討している。マイニングツールの利用の適否は社会的関心事でもあり、この問題を扱った意義は非常に大きい。しかし、用語の理解が十分でない、構成にわかりにくい点等が見受けられた。詳細は、以下のコメントを参考にしていただきたい。

 ・マルウエアは、不正プログラム全般を指すものであり、コンピュータ・ウイルス、ワームなどを含んだ概念です。

 ・crypto-assetを指す語として日本の資金決済法では「暗号資産」としています。判決文中の引用を除き、暗号通貨よりも、暗号資産という語を使用した方がよいと思います。

 ・不正指令電磁的記録の客体該当性が問われた先行裁判例としては、仙台地判平成30年7月2日LEX/DB文献番号25560905、東京高判令和元年12月17日高検速報（令1）号362頁があるので、触れておく必要があるのではないでしょうか。

 ・最高裁は、不正指令電磁的記録の該当性を認める要件として反意図性と不正性を明確に区別することを明らかにし、両者の関係を並列的に捉えていると解されます。この点について、反意図性と不正性の関係について不可分であるという自説からの検討があるとより論旨が明確になると思います。

 ・プライバシー・バイ・デザインは注で説明がある方がよいと思います。また、この点に触れるのであれば、プライバシー・バイ・デザインがGDPRにより法的要求事項になったことや、GDPR違反による高額の制裁金が科された事例が生たことでグローバルスタンダードな設計思想になってきていることを示すとよいのではないでしょうか。

 ・脚注で、法学セミナー、法学教室のみ略称表記となっていますので、他の注同様に正式名とするか、略称で統一するのであれば「法律時報文献月報」を基にするとよいと思います。

 ・構成としては、不正指令電磁的記録に関する罪の概要を示した上で、先行事例の紹介、コインハイブ事件の概要、最高裁判決の分析、反意図性と不正性の要件についての検討とした方が理解しやすくなるように思います。

（該当する場合は要回答）統計分析および解釈は適切ですか。

対象外（統計を使っていない）

本研究は明確かつ正確に提示されたものであり、最新の文献を引用していますか。

はい

方法と分析について第三者による再現が可能となるよう十分な詳細が提示されていますか。

はい

結果の基礎となるソースデータはすべて入手可能で再現性を十全に保証していますか。

はい

結論は結果により妥当な裏付けを得ていますか。

はい

研究設計は適切で学術的価値がありますか。

はい

Reviewer Expertise:

刑法

I confirm that I have read this submission and believe that I have an appropriate level of expertise to confirm that it is of an acceptable scientific standard.

# Response

**Published:** 2022-07-06

**Authors:** Kouya Takara

専修大学 岡田好史 先生

 この度は、お忙しい中、急な査読依頼をお引き受けいただきありがとうございます。

 また、詳細なご指摘をいただきましたことにつきましても御礼申し上げます。

 ご指摘いただきました点を反映させ、近日中に修正版を投稿予定です。

 何卒、よろしくお願いいたします。

 髙良幸哉

# Peer review: Reviewer response for version 1

**DOI:** 10.5256/f1000research.126781.r140928

**Published:** 2022-06-30

**Authors:** Takashi Hikasa

**Affiliations:** 1 Faculty of Management and Information Sciences, Tama University, Tokyo, Japan

　本論文は、コインハイブ事件の最高裁判決を素材に、不正指令電磁的記録に関する罪の、①保護法益、②反意図性、③不正性について検討し、同罪の内実を明らかにするものである。

 　原審では、反意図性に関して「規範的評価」が要求されているものの、最高裁ではこれに関する文言が見受けられない点につき、筆者は、保護法益についての文言部分との対比等を踏まえ、規範的評価自体を否定したとまではいえない旨の指摘をする。かかる指摘は、技術的・社会的背景（CPUの使用程度や消費電力、ウェブサイトにおける広告表示プログラムとの異同）を分析する筆者の根拠づけと相まって、妥当であると考えられる。

 　反意図性および不正性が区分けとして不明確であることやその内容が漠然としていること、両者の関係が独立なのか、反意図性が認められると原則不正性は推定されるのか、など、従来の争点に対し、本論文では、保護法益論（「社会一般の信頼」）との関係性および、クリプトジャッキングの社会的意味を分析することで、これらの問題に対する的確な回答を呈示している。

（該当する場合は要回答）統計分析および解釈は適切ですか。

対象外（統計を使っていない）

本研究は明確かつ正確に提示されたものであり、最新の文献を引用していますか。

はい

方法と分析について第三者による再現が可能となるよう十分な詳細が提示されていますか。

はい

結果の基礎となるソースデータはすべて入手可能で再現性を十全に保証していますか。

はい

結論は結果により妥当な裏付けを得ていますか。

はい

研究設計は適切で学術的価値がありますか。

はい

Reviewer Expertise:

刑法　独占禁止法　サイバー犯罪　AI　自動運転

I confirm that I have read this submission and believe that I have an appropriate level of expertise to confirm that it is of an acceptable scientific standard.

# Response

**Published:** 2022-07-06

**Authors:** Kouya Takara

多摩大学 樋笠尭士 先生

 この度は、お忙しい中、査読依頼をお引き受けいただきありがとうございます。

 近日中に、先生方からのご指摘を反映した修正版を投稿いたします。

 何卒、よろしくお願いいたします。

 髙良幸哉

# Peer review: Reviewer response for version 1

**DOI:** 10.5256/f1000research.126781.r140926

**Published:** 2022-06-27

**Authors:** Osamu Magata

**Affiliations:** 1 Faculty of Law, Chuo University, Tokyo, Japan

**Ⅰ. 本論稿の概要**

 1. ［全体の概略］

 　本論稿は、いわゆる「コインハイブ事件」上告審判決(最判令和4・1・20)を素材に、不正指令電磁的記録に関する罪(刑法168条の2および3)の客体性要件たる「反意図性」および「不正性」について論じたものです。また、本判決に対して批判的な観点から検討が加えられています。

 2. ［筆者の問題意識］

 　これまでの裁判例において、本罪の反意図性要件・不正性要件について詳細に検討したものはなく、これらの要件の認定基準はいまだ明確ではないという点に、筆者の問題意識が向けられているものと思われます。

 3. ［筆者の見解］

 　本罪の保護法益は、一般に、電子計算機のプログラムに対する社会一般の者の信頼という社会的法益であると理解されており、筆者はこれを妥当としています。そのうえで、反意図性要件につき、これも(不正性要件に同じく)法益侵害性と関係する要件であることから、規範的に判断されるものであり、その判断は社会的許容性の観点を入れてなされるべきであるとの主張が示されています。すなわち、反意図性要件は、社会的許容性判断が中心となる不正性要件と「重なり合う」のであり、両要件は「実質的な判断においては区別困難となろう」とするわけです。

 　こうした前提から、本件最高裁判決の結論に関しては同調しつつも、結論を導く論理や事案の特性に関する前提理解において、不十分な点がある旨、指摘がなされています。すなわち「損害が軽微ゆえに、本件プログラムコードの掲載自体の社会的許容性あるとする方が妥当だったのではなかろうか。」、「マイニングプログラムにおいては潜在的なリスクが存する。その点を過小評価しているのではないか」などとの叙述が展開されています。

**Ⅱ. 評価**

 1.［結論］

 条件付き承認

 2.［理由］

 　不正指令電磁的記録に関する罪は、2011年(平成23年)の刑法一部改正により新設された、比較的新しい処罰規定です。そのため、保護法益や成立要件に関する議論は進展過程にあるといえるでしょう。そのような状況のなか、webサイトにアクセスした人の電子計算機のCPUを同意なく使用して暗号資産のマイニングを実行させる行為が、不正指令電磁的記録保管罪(刑法168条の3、168条の２第1項)に当たるとして立件されました。この事案は「コインハイブ事件」として耳目を集めてきましたが、特に問題となったのは、同罪の客体性要件たる「反意図性」・「不正性」の充足いかんです。上記最高裁判決は、この争点に関して一定の判断を示し、無罪との結論を示したものであり、その意義は大きいといえます。

 　筆者、高良氏による本論稿は、そのような新しい重要な最高裁判断を扱い、分析したものである点で、少なくない意義があります。また、反意図性要件および不正性要件をめぐる解釈のあり方について検討がなされているとともに、ドイツにおける類似事案の取扱いについての言及もあり、参照価値があります。さらに、反意図性要件および不正性要件に関し、実質判断において区別し難いものであり、重なり合う要件であるとする筆者の主張は興味深く、議論の活性化を促しうるものといえます。

 　もっとも、いくつかの点で不明確な点が見受けられるため、条件付き承認と判定します。

 ・全体を通しての留意点は、「Ⅲ. コメント」として記載します。参考になさってください。

 ・具体的な要修正箇所は、「Ⅳ. 要修正箇所」(1)-(9)として記載します。これらに関しては、適切に修正を行ってください(承認条件)。

 ・修正は必須ではありませんが、参考にしていただきたい点を「Ⅴ. 参考意見」(a)-(d)として挙げます。

**Ⅲ. コメント** **(** **全体を通しての留意点)**

 　筆者によれば、本稿は「コインハイブ事件の各裁判例を分析・検討することを通して、不正指令電磁的記録に関する罪における反意図性と不正性の判断基準を明らかにすることを目的とするもの」であるかことから、論文として執筆されたものと思われます。

 　しかし、反意図性要件・不正性要件それぞれの内容に関する筆者自身の見解の全体像が、必ずしも明確に叙述されていません。その意味で、反意図性・不正性の判断基準を明らかにするという筆者の目的が達せられていないきらいがあります。両要件の内容をいかに解するのかは本論文のコアとなる部分ですから、より充実し、整理された論述が求められます。

**Ⅳ. 要修正箇所**

 (1) Ⅲ・2・第4段落: 「情報リテラシーについては利用者において差があり、当該プログラムについて個々人としてみた場合、例えばエンジニアにおいて一般的に知りうる情報を、情報機器に疎い者はもちろん通常のユーザーですら知りえないことは当然にあることである。この点、日々開発されるプログラムについて通常人においては知りえず、常に「一般的に認識すべき」場合ではなく、反意図性が認められうるのであり、一般人の認識可能性のみを基礎とするのであれば、反意図性の範囲は規範的判断によって限定した意味を失してしまうであろう。」の部分について。

 　　(1-1)「限定した意味を失してしまう」は、過度な否定的評価と思われます。仮に、一般人の認識可能性のみを基準としたとしても(一般人の認識可能性と実際との形式的な齟齬のみから反意図性を判断したとしても)、たとえば、プログラム送信を受けた実際のユーザーは情報機器に疎かったことから認識できかったものの一般ユーザーならば認識することができたといえるプログラムの動作が問題となった場合に、反意図性を規範的に否定できるのですから、規範的判断による限定機能はその意味で有効に働きます。「限定した意義が弱まってしまう」など、少なくとも表現を変えることを含め、検討してください。

 　　(1-2)おそらく筆者は、高度な知識をもつ特別なユーザーのみが知りうるプログラムが問題となるケース(一般ユーザには認識できないケース)であっても、場合によっては、反意図性を否定すべきだと考えるのでしょう。しかし、どういう場合がそれに当たるのかについて具体的な叙述がなく、主張が伝わりにくくなっています。この部分について加筆等を行ってください。

 　(筆者の立場はおそらく控訴審が展開した論理に近いにように思われるので、加筆に当たってはその点にも留意するとよいでしょう。)

 (2) Ⅲ・2・第4段落:「仮に「一般人に認識すべき」プログラムであったとしても、当該プログラムが多大な負担を強いるのであれば、一般人がこれを望まないことは、当然にありうる。かかる判断においては、実質的な負担の存否・軽重などに鑑み、どの程度の負担であれば一般に許容できるのか、つまりは、社会的許容性を観念せざるを得ないと思われる。」の部分について。

 　　(2-1)「多大な負担」、「実質的な負担」、「どの程度の負担」というように「負担」という語が多用されていますが、用語として適切か再検討し、より適切な表現に変えてください。

 　　(2-2) 筆者は、「どの程度の負担であれば一般に許容できるのか」という(社会的許容性の)観点を、反意図性の要件判断において(も)用いるべきであるとしています。この観点は、(本件最高裁判断が示すように、)もっぱら不正性要件に関わるものとして位置づけることも十分可能であるため、なぜ反意図性の要件に(も)かかわらしめるのかについて、より立ち入った説明が求められます。

 (3) Ⅲ・2・第5段落:「両者は重なりあうものであり、形式的な分類はともかくとして、実質的な判断においては区別困難となろう。」の部分について。

 　　(3-1) 両者(反意図性要件と不正性要件)が「重なり合う」とは、より詳しくはどういうことか、またなぜそうなるのか、もう少し説明を付してください。

 　　(3-2) また、両者は「実質的な判断において区別困難となろう」としていますが、これは、そうなってしかるべきだ(本来そういうものだ)という趣旨であるのか、そうであってはならないという意からのものか、判然としません。他の叙述部分から推測しますに、おそらく前者に近いものと思われますが〔Ⅳ・「不正性について」の部分に「反意図性と不正性は不可分であるとの私見」とある〕、やはり主張の趣旨が明確でないので、書きぶりを改善することを検討なさってください。

 (4) Ⅲ・2・第6段落:

 　(4-1) この段落において「意図」という語が複数回用いられていますが、行為者の主観面としての意図と、一般ユーザーの規範的主観面としての意図とを、同じ扱いにしているように読めます。このような書き方でよいのか再検討なさってください。

 (5) Ⅳ・「反意図性について」: 「反意図性については、「一般の使用者が認識すべき動作と実際の動作が異なる場合に肯定される」とし、一般の認識可能性を判断基準としつつ、文言上は一般人の認識と現実の齟齬を判断している事実的判断であるようにも見える。」の部分について。

 　　(5-1)「一般人の認識と現実の齟齬を判断」する手法を「事実的判断」と呼ぶのは、ミスリードとなりえます。具体的な個々のユーザーの実際の認識でなく、通常ユーザーの認識可能性というものを想定し、それと現実との齟齬の有無を判断基準としようとしている時点で、すでにそれは規範的判断です。「事実的判断」ではなく、異なる表現を用いることを検討してください。

- ひょっとすると、筆者において、「規範的判断」・「事実的判断」の意について誤解があるかもしれませんので、以下、参考までに若干説明しておきます。
- 反意図性要件をめぐって使用される「規範的判断」という語は、二通りに使用されています。すなわち、第一は、個別具体的な使用者の実際の認識を基準とするのではなく、一般に認識すべきと考えられるところを基準にするという意味〔一般人であれば知りうるプログラムの動作かという観点〕として使用されており、第二は、当該プログラムの機能の内容や機能に関する説明内容、想定される利用方法等を考慮に入れて、一般ユーザーであれば許容するプログラムの動作かどうかを基準にするという意味〔一般人基準で見たときの当該プログラムの許容可能性〕として使用されています。
- 1審も最高裁も、一般人の認識可能性を問題にし、それと現実との齟齬の有無を基準にする判断手法に依拠していますので、その限りでやはり規範的判断(第一の意味における規範的判断)を展開しているといえます (ただ、第二の意味における規範的判断はしておらず、その点で控訴審の判断方法と相違がありますので、そうしたところを押さえた論評をすべきでしょう)。

　　(5-2) 如上の観点からしますと、「本件最高裁が反意図性の判断における規範的判断を放棄したとまではいえないであろう。」という分析内容は不可解に映ります。この部分の叙述についても再検討なさってください。

 (6) Ⅳ・「不正性について」: 「この点、反意図性と不正性は不可分であるとの私見によれば妥当な判断であると思われる。」の部分について。

 　　(6-1) 反意図性と不正性が「不可分」とはどういう趣旨であるか、説明を付してください。

 (7) Ⅳ・「不正性について」:「しかし、同意を得ずに他者のコンピュータに介入操作するクリプトジャッキングの潜在的な危険性に鑑みれば、「社会一般の信頼」を害する危険性は少なくない。」の部分について。

 　　(7-1) そうなのであれば、最高裁の結論とは異なり、不正性を肯定すべきではないかとの疑問が生じます。この点、筆者は、「損害が軽微ゆえに…社会的許容性〔が〕ある」と説いていますが、何をもって損害が軽微であるというのか、説明が欲しいところです。この点について説明を付してください。

 (8) Ⅴ・第2段落: 「結びに変えて、本稿で扱ったコインハイブ事件の射程と意義を述べたい。」の部分について。

 　　(8-1) 本判決の意義は述べられていますが、射程について実質的な明確な記述が見当たりません。本判断は具体的にどういう事案に判断拘束性を及ぼすのか、言及してください。

 　(なおその際、本件が、閲覧者の電子計算機のCPU使用率調整値を0.5と設定していた事案であることにも留意するとよいでしょう。)

 (9) Ⅴ・第2段落: 「反意図性が認められる場合の処罰範囲を限定するものとして不正性の要件があり、反意図性判断が不正性判断の前段階におかれることが明示された点は評価できる。」の部分について。

 　　(9-1) 上記は、本件最高裁判断に対する筆者の評価ですが、筆者はⅣ・「不正性について」のなかで、「反意図性と不正性とは不可分である」と主張しており、「評価できる」の趣旨が必ずしも明らかではありません。いかなる趣旨であるのか述べてください。

**Ⅴ. 参考意見**

 (a) Ⅲ・2・第2段落・最後の一文

 　　(a-1)伝えたいことが伝わる文になっているか再検討してください。

 (b) Ⅲ・2・第4段落:「仮に「一般人に認識すべき」プログラムであったとしても、当該プログラムが多大な負担を強いるのであれば、一般人がこれを望まないことは当然にありうる。かかる判断においては、実質的な負担の存否・軽重などに鑑み、どの程度の負担であれば一般に許容できるのか、つまりは、社会的許容性を観念せざるを得ないと思われる。」の部分について。

 　　(b-1) 話題はここで、直前までの叙述内容(一般人に認識できないプログラムの話題)とは逆のケース、すなわち一般人が認識すべきプログラムの話題に切り替わっていますが、書きぶりからそうだとは分かりにくく、読者を混乱させる恐れがあります。上記部分の直前に、「反対に」とか、「逆に」など、適切な接続詞を入れるとよいように思われます。

 　　(b-2) 筆者は、「どの程度の負担であれば一般に許容できるのか」という(社会的許容性の)観点を、反意図性の要件判断において(も)用いるべきであるとしています。しかし、そうすると、すでに論者によって指摘されているように(永井善之「判批」新・判例解説 Watch 刑法 No.176、4頁参照)、反意図性要件の判断は不正性要件の判断の先取りまたはそれとの一体化に至りうるかもしれません。筆者はこの点についてどう考えるでしょうか。読み手が関心をもつこととなりうるので、可能であればこの点に言及されると、説得性が増すでしょう。

 (c) Ⅳ・「不正性について」:「不正性については、原審において「閲覧者に利益を生じさせない一方で一定の不利益を与えるものである上、不利益に関する表示等もされない」点で、「閲覧者の電子計算機を、閲覧者以外の利益のために無断で使用する点を基礎に不正性、本罪の保護法益である、「社会一般の信頼」保護という原則により依拠したものであるといえる。」の部分について。

 　　(c-1) 読み手に伝わる表現になっているか、確認なさってください。

 (d) Ⅴ・第2段落: 「私論〔試論?〕であるが、裁判所が反意図性判断における規範的判断を放棄していないと考えるのであれば、(1) 反意図性にかかる事実判断、(2) 反意図性にかかる規範的判断、(3) 不正性判断との流れで検討がなされ、処罰範囲を限定していくとも考えられる。」の部分に関して。

 　　(d-1) (前述のように、)そもそも、裁判所が反意図性判断における規範的判断を放棄しているとは思えないので、「放棄していないと考えるならば」という論の進め方には違和感があります。

 　　　　　　　　　　　　　　　　　　　　　　以上

（該当する場合は要回答）統計分析および解釈は適切ですか。

対象外（統計を使っていない）

本研究は明確かつ正確に提示されたものであり、最新の文献を引用していますか。

一部該当

方法と分析について第三者による再現が可能となるよう十分な詳細が提示されていますか。

はい

結果の基礎となるソースデータはすべて入手可能で再現性を十全に保証していますか。

はい

結論は結果により妥当な裏付けを得ていますか。

一部該当

研究設計は適切で学術的価値がありますか。

はい

Reviewer Expertise:

法学・刑事法

I confirm that I have read this submission and believe that I have an appropriate level of expertise to confirm that it is of an acceptable scientific standard, however I have significant reservations, as outlined above.

# Response

**Published:** 2022-07-06

**Authors:** Kouya Takara

中央大学 曲田統 先生

 この度はお忙しい中、査読依頼をお引き受けいただきありがとうございます。

 また、詳細なご指摘をいただきました点につきましても御礼申し上げます。

 ご指摘いただきました点を反映し、近日中に修正版を投稿いたします。

 何卒よろしくお願いいたします。

 髙良幸哉
